# Supplementary material for: Necroptosis as a Novel Facet of Mitotic Catastrophe
Source: Int J Mol Sci. 2022 Mar 29;23(7):3733. doi: 10.3390/ijms23073733 (PMC8998610; doi:10.3390/ijms23073733)
Supplement: Supplementary file 1 [file ijms-23-03733-s001.zip › ijms-1591295-supplementary.pdf]

Supplementary Figures

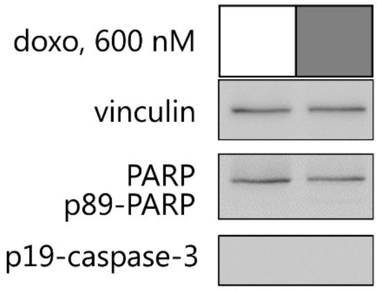

Figure S1. Doxorubicin treatment (600 nM) of non-tumor human kidney cell line HEK293T. Immunoblot analysis using the indicated antibodies is shown.

A

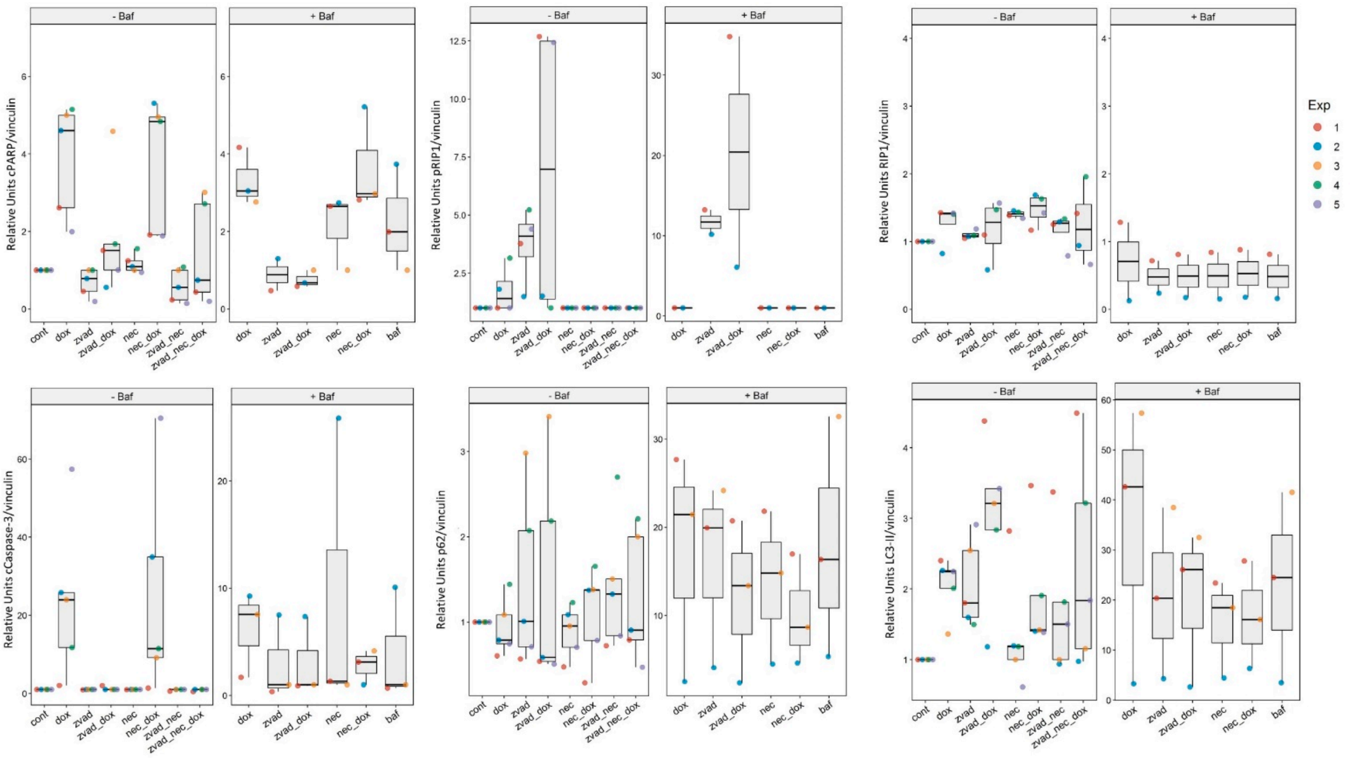
- Baf
+ Baf
Relative Units cPARP/vinculin
Relative Units pRIP1/vinculin
Relative Units RIP1/vinculin
Relative Units cCaspase-3/vinculin
Relative Units p62/vinculin
Relative Units LC3-II/vinculin
cont
dox
zvad
zvad_dox
nec
nec_dox
zvad_nec
zvad_nec_dox
baf
Exp
1
2
3
4
5

B

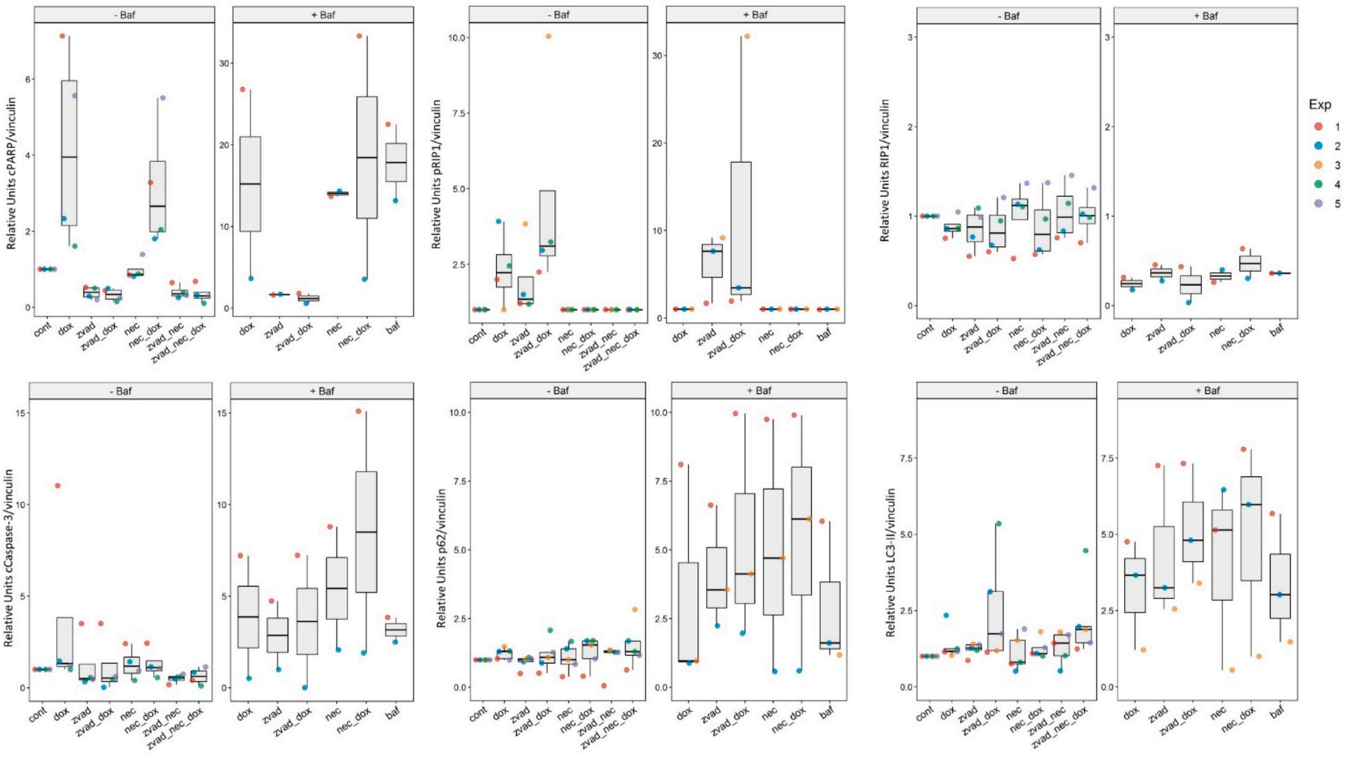
- Baf
+ Baf
Relative Units cPARP/vinculin
Relative Units pRIP1/vinculin
Relative Units RIP1/vinculin
Relative Units cCaspase-3/vinculin
Relative Units p62/vinculin
Relative Units LC3-II/vinculin
cont
dox
zvad
zvad_dox
nec
nec_dox
zvad_nec
zvad_nec_dox
baf
Exp
1
2
3
4
5

C

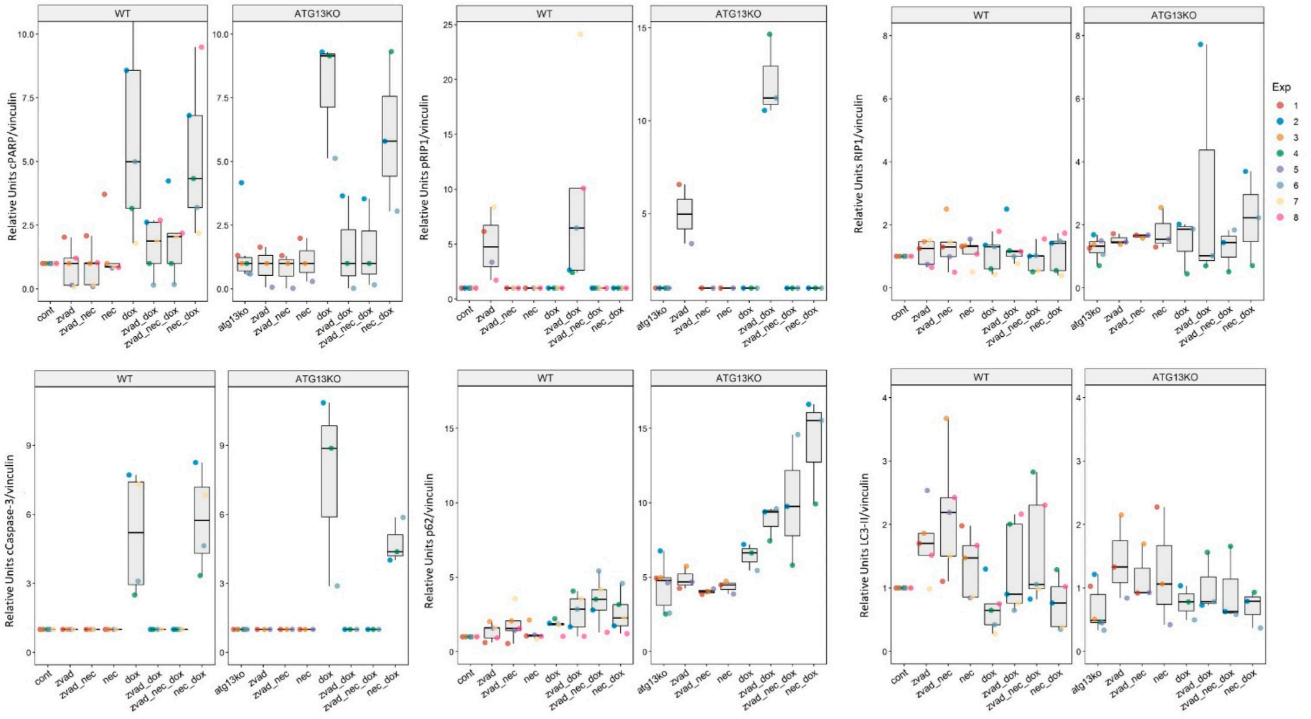

D

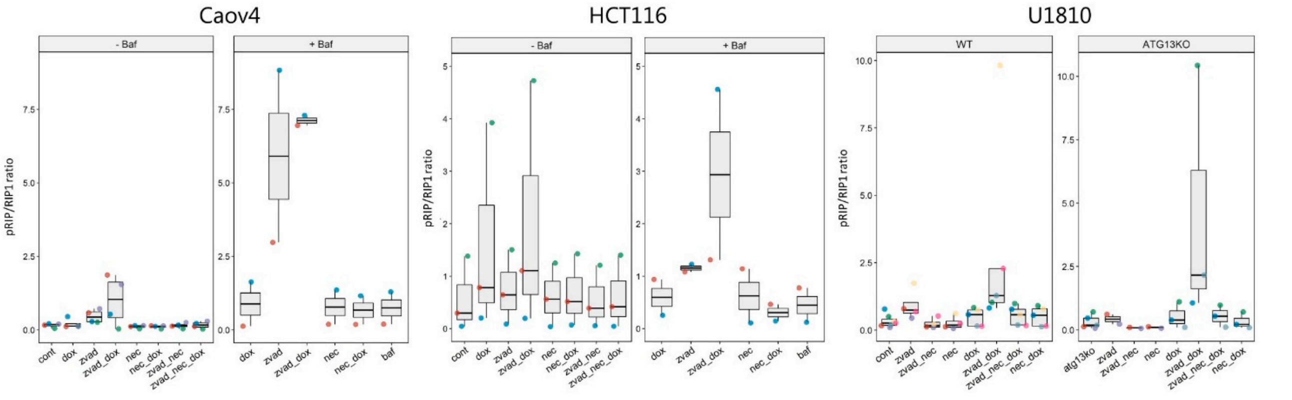

Figure S2

Immunoblot quantification of p19-caspase-3 (cCaspase-3), p89 PARP (cPARP), p62, LC3-II, pRIP1, RIP1 protein levels normalized to vinculin level in (A) Caov4, (B) HCT116, (C) U1810 cell lines and (D) pRIP1/RIP1 ratio presented by boxplot graphs. All values are in relative units. Each boxplot includes values from several different experiments (Exp).

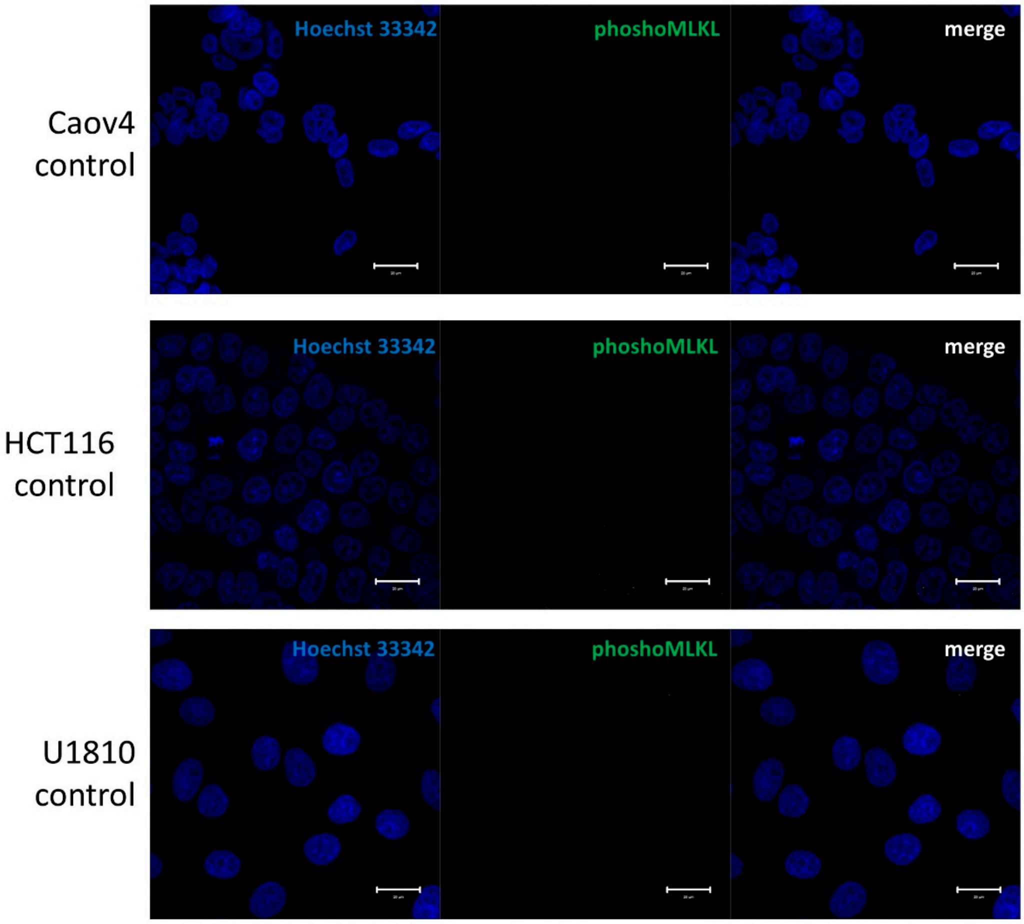

Figure S3. Immunofluorescence analysis using anti-phospho-MLKL antibodies in Caov4, HCT116, and U1810 untreated cells. Cell nuclei were counterstained with Hoechst 33342 (blue). Scale bars: 10 mkm.

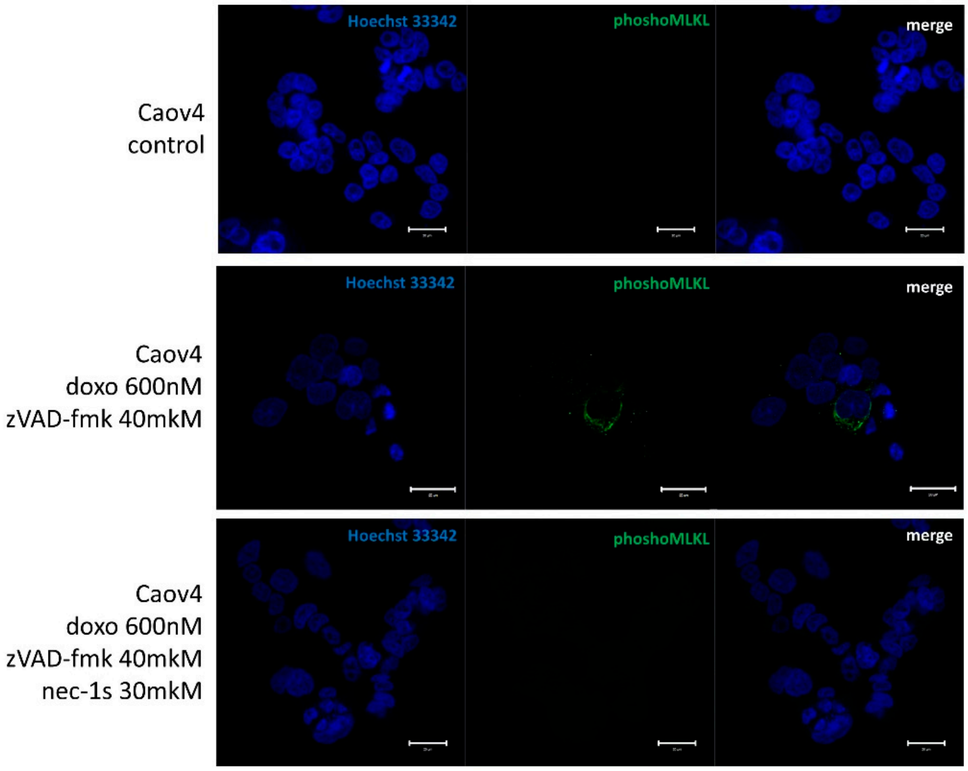

Figure S4. Immunofluorescence analysis using anti-phospho-MLKL antibodies in Caov4 cells treated with the indicated agents. Green dots indicate phospho-MLKL. Cell nuclei were counterstained with Hoechst 33342 (blue). Scale bars: 10 mkm.
